# A Case of Typhoid Fever, in Which the Hydriodale of Potassa Was Successfully Administered

**Published:** 1845-12

**Authors:** C. B. Voigt


*A Case of Typhoid Fever, in which the Hydriodate of Potassa was successfully administered.* By C. B. Voigt, M. D.

This case occurred in the month of June of the present year. The subject was a young man, 27 years of age, of the refractory constitution, fair ordinary health, and accustomed to a laborious occupation. The symptoms, when he came under my notice, were headache, suffused eyes, a dull or stupid countenance, somnolency, flushed and heated surface, and a full, irritated and frequent pulse. The tongue was contracted, reddened, and coated with a dark brown fur. There was considerable sensitiveness of surface, apparently neuralgic, over the right hypochondrium; tenderness on pressure in the right iliac region; and a diarrhœa of thin and frequent evacuations. The latter continued through the whole course of the disease, and, together with the local tenderness and other symptoms, led to the suspicion that not mere enteric irritation, but, probably, follicular inflammation, was also an attendant feature of this case.

Towards the evening of the seventh day of the attack, the hydriodate of potassa was prescribed, with a view to its tonic and alterative properties. At this time the strength of the patient was seriously reduced: he was still somnolent and delirious. The diarrhœa, though somewhat diminished, was not entirely overcome; and the tongue was still contracted, red and furred. Some tenderness to pressure still existed in the right iliac region. (For this he had been cupped in an earlier part of the treatment.) And there were a few small vesicles on the upper lip. As exhaustion was impending, and fearing a farther loss of strength, I directed ℈ij of the hydriodate of potassa in four ounces of mucilage, of which two teaspoonsful were to be given every three hours. Three drops of laudanum were added to each dose of of the medicine, in reference to the diarrhœa, and in place of a chalk mixture, containing gentle anodynes and a small quantity of the bals. copaiba, previously in use.

By the time I saw him next morning he had taken four doses of this medicine, and I found him highly improved, although the diarrhœa was freer than on the evening previous. His fever now was slight: he was stronger; and somnolency, stupor and delirium had disappeared.. His tongue was fuller and improved in colour, and the eruption about the mouth presented a new aspect. In addition to the soreness on the upper lip, to which it was at first confined, it now extended from the angle of the mouth in a semicircular form to the corner of the nose, and in the course of the day assumed an appearance resembling the eruption of small pox when about to become pustular. The chalk mixture was again resumed, and a teaspoonful of the hydriodate medicine allowed every four hours. At noon of this day—the eighth of the attack—fever had entirely subsided; and on the evening of the ninth, the patient being well enough, he was discharged from further medical treatment.

