# Errata

**Published:** 1846-01

**Authors:** 


ERRATA.

All who write for the press are compelled at times to feel the mortification of seeing in their productions, when issued from the press, letters added or omitted, and sometimes even words and phrases left out, or put in a connection which entirely alters or obscures the meaning designed to be conveyed. This is eminently our own experience; and yet we rarely make any subsequent correction; not that it is not required, but that we prefer trusting to our readers to correct any little deficiencies, rather than presume that they are incapable of it. Still cases will occur in which, from the nature of the subject, the reader may not be able to make out the author's meaning from the context, as in the instances referred to in the following note:

Dear Sir:—If it can be conveniently done, I would esteem it a favour for the following errata, relating to the case of mine published in the last No. of the Examiner, to be noticed in the No. forthcoming. The first is an error in the print: the second an omission of my own—the effect of haste and a multiplicity of private concerns at the time the copy was written.

In the first paragraph, last line but one, the print reads, "that not mere enteric irritation, but, probably, follicular inflammation, was also an attendant," &c. It should read thus: that enteric irritation—perhaps follicular inflammation—was also an attendant feature of this case. In the third paragraph, second line from the foot of the page, (715,) the following note should have been inserted as an appendage to the word pustular: *I supposed this eruption to be a form of eczema.

Very respectfully,
C. B. Voigt.

Prof. Huston.
Dec. 18th, 1845.

